# Dieselzymes: development of a stable and methanol tolerant lipase for biodiesel production by directed evolution

**DOI:** 10.1186/1754-6834-6-70

**Published:** 2013-05-07

**Authors:** Tyler P Korman, Bobby Sahachartsiri, David M Charbonneau, Grace L Huang, Marc Beauregard, James U Bowie

**Affiliations:** 1 Department of Chemistry and Biochemisty, UCLA-DOE Institute of Genomics and Proteomics, Molecular Biology Institute, University of California, Los Angeles, USA; 2 Département de chimie-biologie, Université du Québec à Trois-Rivières, Trois-Rivières, Québec, Canada

**Keywords:** Lipase, Biodiesel, *Proteus mirabilis*, *Proteus sp. K107*, Directed evolution, Alcohol tolerance

## Abstract

**Background:**

Biodiesels are methyl esters of fatty acids that are usually produced by base catalyzed transesterification of triacylglyerol with methanol. Some lipase enzymes are effective catalysts for biodiesel synthesis and have many potential advantages over traditional base or acid catalyzed transesterification. Natural lipases are often rapidly inactivated by the high methanol concentrations used for biodiesel synthesis, however, limiting their practical use. The lipase from *Proteus mirabilis* is a particularly promising catalyst for biodiesel synthesis as it produces high yields of methyl esters even in the presence of large amounts of water and expresses very well in *Escherichia coli*. However, since the *Proteus mirabilis* lipase is only moderately stable and methanol tolerant, these properties need to be improved before the enzyme can be used industrially.

**Results:**

We employed directed evolution, resulting in a *Proteus mirabilis* lipase variant with 13 mutations, which we call Dieselzyme 4. Dieselzyme 4 has greatly improved thermal stability, with a 30-fold increase in the half-inactivation time at 50°C relative to the wild-type enzyme. The evolved enzyme also has dramatically increased methanol tolerance, showing a 50-fold longer half-inactivation time in 50% aqueous methanol. The immobilized Dieselzyme 4 enzyme retains the ability to synthesize biodiesel and has improved longevity over wild-type or the industrially used *Brukholderia cepacia* lipase during many cycles of biodiesel synthesis. A crystal structure of Dieselzyme 4 reveals additional hydrogen bonds and salt bridges in Dieselzyme 4 compared to the wild-type enzyme, suggesting that polar interactions may become particularly stabilizing in the reduced dielectric environment of the oil and methanol mixture used for biodiesel synthesis.

**Conclusions:**

Directed evolution was used to produce a stable lipase, Dieselzyme 4, which could be immobilized and re-used for biodiesel synthesis. Dieselzyme 4 outperforms the industrially used lipase from *Burkholderia cepacia* and provides a platform for still further evolution of desirable biodiesel production properties.

## Background

Biodiesel is a well-validated transportation fuel that is an attractive alternative to petrodiesel [1]. Biodiesel burns cleaner, releases less CO_2_ to the atmosphere, is biodegradable and can be obtained from renewable sources [1,2]. Moreover, biodiesel can be used in existing diesel engines and is compatible with current fuel distribution infrastructure. Finding new sources of biodiesel and improved production methods is therefore an important goal in achieving diversification of energy resources [2].

**Figure 1:**
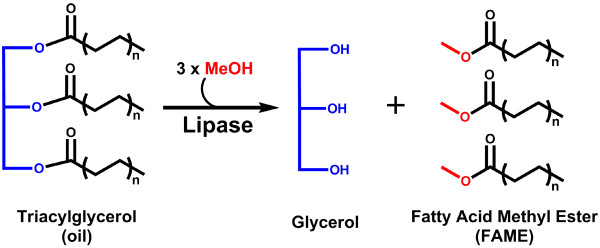
Schematic of the general transesterification reaction catalyzed by lipases.

Biodiesel is typically composed of a mixture of fatty acid methyl esters (FAME) (Figure 1), although other esters can be used [3]. Biodiesel is usually synthesized via the base-catalyzed transesterification of triacylglycerol (TAG) oils with methanol (MeOH) or ethanol (EtOH). In the base-catalyzed reaction, the presence of water leads to the production of free fatty acids (FFAs), which are a dead-end product. Moreover, any FFAs present in the oil cannot be converted to methyl esters. FFA contamination requires additional purification and also leads to emulsions that complicate reaction clean up. In addition, glycerol, the other value-added reaction product, becomes contaminated with salt and base, increasing purification costs and lowering its value [1].

Lipases are a family of enzymes that are interesting as an alternative catalyst for biodiesel production [4]. Like chemical catalysts, lipases can catalyze the transesterification of TAG with short chain alcohols at significant rates. However, unlike the base-catalyzed reaction, some lipases can also convert FFAs to FAMEs even in the presence of high levels of water [4,5]. Moreover, the lipase catalysts can be easily separated from the glycerol product, thereby eliminating glycerol contamination and increasing its potential as a value-added product to lower the net cost of biodiesel production [6,7].

An obvious drawback to the use of lipases is the high cost of the catalyst relative to a simple base such as NaOH [7,8]. The initial investment cost of a lipase catalyst could be greatly mitigated, however, if it could be used for long periods of time to generate large quantities of biodiesel. Unfortunately, the presence of high concentrations of MeOH used for biodiesel synthesis can cause rapid loss of lipase activity due to alcohol-induced inactivation [9].

Various approaches have been employed to increase lipase lifetimes for biodiesel synthesis [10]. Process engineering approaches have included enzyme immobilization, the use of co-solvents, or the step-wise feeding of alcohol so the concentration remains low [11]. However, the use of co-solvents further increase costs while step-wise feeding of MeOH is difficult on an industrial scale. In addition, there have been efforts to identify natural lipases that are inherently more tolerant of the harsh conditions employed for biodiesel production [12]. To our knowledge, rational design or directed evolution approaches have not been used to improve natural lipases specifically for this non-natural role.

Directed evolution and rational design of existing lipases have the potential to produce long lasting re-engineered enzymes with specific properties [13]. For example, previous reports have shown that many α/β hydrolase fold enzymes, including lipases, have been successfully reengineered by directed evolution for improved thermostability as well as tolerance to a variety of polar solvents such as dimethylsulfoxide (DMSO), dimethylformamide (DMF), and acetonitrile (MeCN) [14-18]. To date, there are no reports of the reengineering of a lipase specifically for improved tolerance to short-chain alcohols such as MeOH and EtOH. The development of a lipase highly resistant to MeOH inactivation would be especially useful as a catalyst for the economical synthesis of biodiesel.

Many microbial lipases have been identified as potential enzyme catalysts for biodiesel synthesis with a wide range of stabilities and catalytic efficiency [12]. In spite of the large number of identified microbial lipases, most are poor targets for directed evolution methods because they require chaperones and/or post-translational modifications specific to the host organism [19]. As a result most microbial lipases can only be produced in their native host, hampering engineering efforts. As an example, one of the most widely used industrial lipases, LipA from *Burkholderia cepacia*, is highly active and tolerant to short-chain alcohols but requires a chaperone for proper folding and does not express solubly in *Escherichia coli*[19].

Recently, an effective biodiesel-producing lipase from *Proteus sp.* K107 was identified that can be expressed solubly at high levels in *E*. *coli*[20]. The *Proteus sp*. K107 lipase is 100% identical to a lipase from *Proteus mirabilis* so we will refer to it as *Proteus mirabilis* lipase (PML). PML belongs to the *Proteus*/psychrophilic subfamily of I.1 lipases which lack a leader sequence and a disulfide bond present in other family I.1 and I.2 lipases [21]. PML was shown to be tolerant to short-chain alcohols such as MeOH and EtOH and to catalyze the synthesis of fatty acid methyl esters (biodiesel) in the presence of high concentration of water. Highlighting the usefulness of PML as a potential low-cost catalyst for biodiesel production [22], a recombinant *E*. *coli* strain overexpressing PML could also be used as a whole-cell biocatalyst for FAME synthesis [20]. Unfortunately, PML is irreversibly inactivated by incubation at over 50% MeOH, a common weakness of lipases [23]. In this paper we report the development of a PML variant with significant increases in thermostability and tolerance to MeOH that retains nearly wild-type activity at ambient temperature. A high-resolution crystal structure of the optimized lipase provides insight into adaptations in PML leading to heat and MeOH tolerance.

## Results and discussion

### Dieselzyme 1: generation of disulfide mutant increases stability

**Figure 2:**
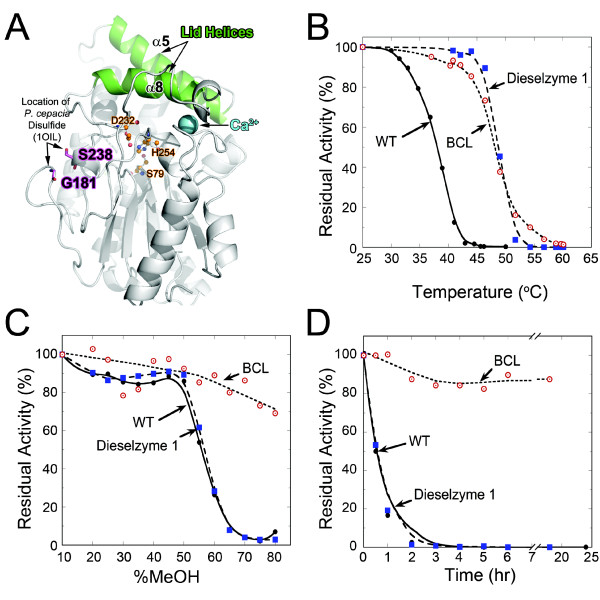
**Effect of introduced disulfide bond on thermal and methanol induced inactivation.** (**A**) Structure of *P. mirabilis* lipase. Lid helices are shown in green, bound Ca^2+^ in blue, and catalytic triad as orange ball and stick. The location of the equivalent disulfide bond found in the *B. cepacia* lipase but absent in PML is labeled and shown as sticks. (**B**) Thermal inactivation as a function of temperature. Residual activity was measured after incubation for 1 hour. (**C**) Inactivation as a function of methanol concentration. Residual activity was measured after incubation for 2 hours after dilution to 10% methanol. (**D**) Time course of inactivation by 50% methanol. Samples were diluted to 10% methanol prior to assaying for residual activity. Results are the average of three independent experiments. Error-bars are omitted but are less than 5% in all cases. Lines are shown for clarity and represent the best-fit to the data points. WT PML(black circles), BCL (red open circles), and Dieselzyme 1 (blue squares).

The introduction of disulfide bonds is a common strategy to improve enzyme stability that has successfully been applied to other lipases [24,25]. Based on sequence analysis, we hypothesized that we could stabilize the PML by the introduction of a disulfide bond. Homologous lipases from *Pseudomonas aeruginosa* and *Burkholderia cepacia* (42% and 38% ID to PML respectively) contain a single disulfide bond between residues 181 and 238 (PML numbering) that is not conserved in PML [26,27]. The PML wild-type structure showing the location of the corresponding disulfide bond in *B*. *cepacia* (1OIL) can be seen in Figure 2A. Due to the structural conservation of this region and the proximity of G181 and S238 in PML, a disulfide bond was introduced between residues G181 and S238 by mutation to cysteines (Figure 2A). We refer to this mutant as Dieselzyme 1.

The effect of the introduced disulfide bond on resistance to thermal inactivation was determined by monitoring the residual lipase activity after heat treatment. Samples were incubated for 1 hour between 37 and 60°C and the residual activity was measured at 25°C. As shown in Figure 2B, the introduction of a single disulfide in Dieselzyme 1 significantly stabilized PML, increasing the half-inactivation temperature (IT_1/2_) from 37°C for the wild-type enzyme to 48°C for the Dieselzyme 1. Also, when incubating at a constant 50°C, the half-life increased from less than 15 min for wild-type to ~75 min for Dieselzyme 1 (Additional file 1: Figure S1). Interestingly, Dieselzyme 1 displays the same temperature inactivation profile as the native *Burkholderia cepacia* lipase (BCL) which contains a single disulfide in the same position (Figure 2B, Additional file 1: Figure S1). Analysis of the crystal structure (below) shows clear electron density for the disulfide bond (Additional file 2: Figure S2A) suggesting the improvement in thermostability is a direct result of disulfide bond formation.

While thermostability is an important property that contributes to the industrial usefulness of an enzyme, a more thermostable enzyme does not necessarily mean that an enzyme will be more tolerant to organic solvents, especially relatively polar, water miscible solvents such as methanol and ethanol that are used in biodiesel synthesis. Indeed, work with *Pseudomonas fragi* lipase has shown that increasing thermostability does not necessarily lead to improved solvent tolerance [28]. Additionally, many thermostable lipases such as the *Thermomyces lanuginosus* lipase display only modest alcohol tolerance [29]. The effect of methanol on wild-type PML and Dieselzyme 1 was monitored by measuring the residual activity after incubation with various concentrations of methanol for 2 hours at 25°C. The introduced disulfide bond in Dieselzyme 1 does not have an effect on the methanol tolerance of PML as the methanol inactivation profiles for wild-type PML and Dieselzyme 1 are identical (Figure 2C and 2D). Thus, consistent with earlier work, it appears that the mechanism of methanol inactivation is different from heat inactivation. We therefore employed directed evolution to improve the methanol tolerance Dieselzyme 1.

### Dieselzymes 2–4: directed evolution

**Figure 3:**
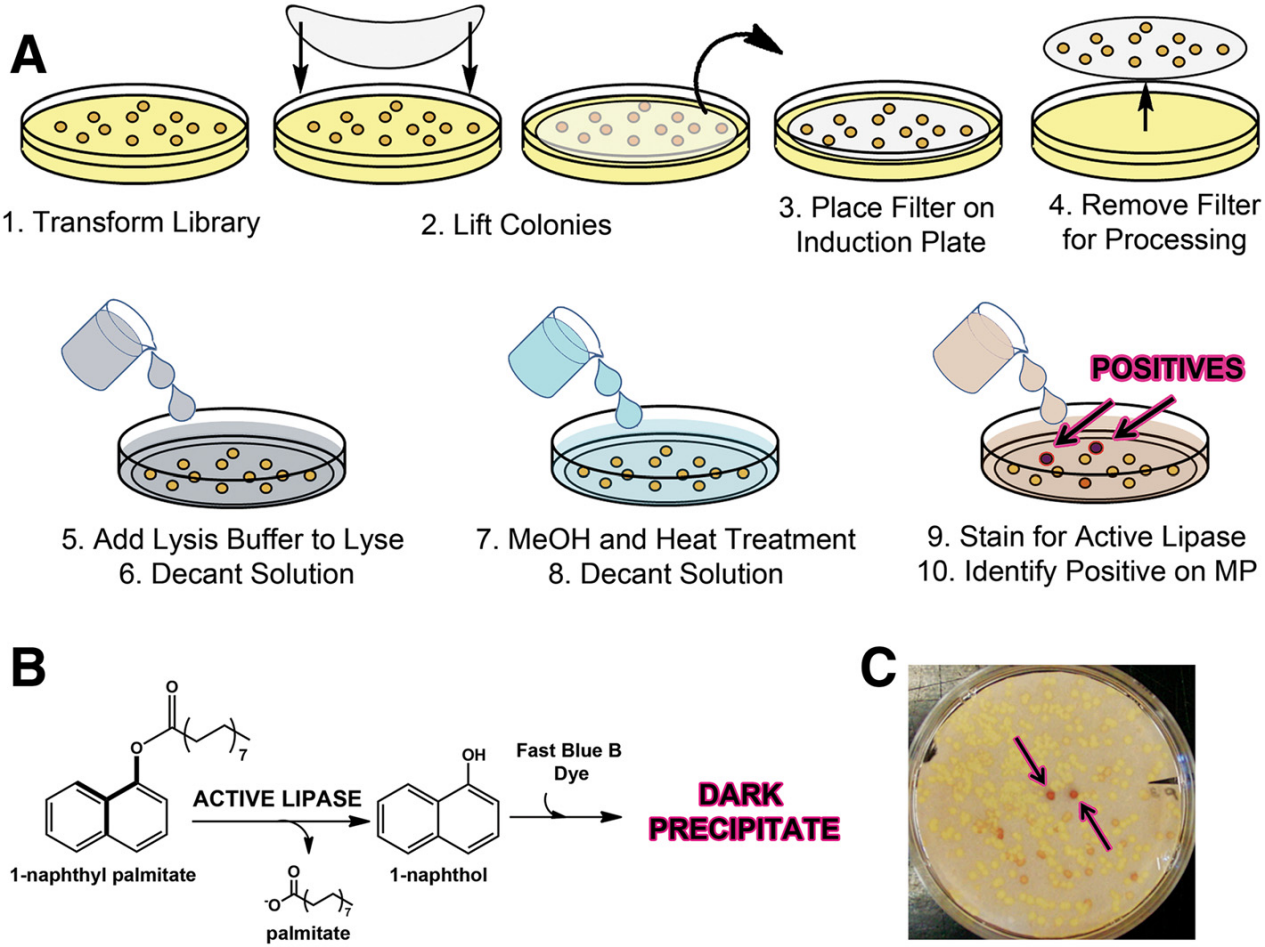
**Schematic of the screening protocol for improved methanol tolerance by directed evolution of PML.** (**A**) Steps for colony lift assay and staining protocol. (**B**) Hydrolysis of 1-napthyl palmitate and detection of 1-naphthol by fast blue to assay colonies for residual activity after exposure to methanol and heat. (**C**) A representative filter following step 9 in (**A**). Black arrows point to potential positives identified on the filter.

To screen for mutants with improved methanol and heat tolerance, we developed a rapid colony assay for stability screening (Figure 3). Following error-prone PCR, mutant library colonies were lifted onto filter paper and protein expression was induced by placing the colonies face up on LB-agar containing 1 mM IPTG for 3 hours. The lipase variants expressed in each colony were then tested for their resistance to inactivation by incubating the filters in heat and methanol. After removal of the methanolic incubation solution, mutants that retained significant residual activity could be identified by overlaying with a solution of 1-naphthyl palmitate and Fast Blue B dye suspended in agar, leading to the formation of a dark purple/brown colony when active lipase is present.

To screen for improved methanol tolerance, we started with the stable disulfide mutant Dieselzyme 1 as the parent for error-prone PCR. In the first round, the mutant library was incubated with 50% methanol at 45°C for 30 minutes prior to screening for residual activity. In the second round, the mutant library was incubated with 65% methanol at 45°C for 45 minutes. Finally, the third round of screening was performed in 65% methanol at 47°C for 90 minutes. During each round of screening, only the mutant clones displaying residual activity within the first ten minutes were picked and confirmed in a second colony screen as above. Typically, 10–15 mutants out of ~20,000 were selected for further validation during each round of screening.

The mutant lipases positive for increased methanol resistance after each round were purified and tested for their tolerance to methanol by measuring residual activity after a 16 hr incubation in 70% methanol at 25°C. Beneficial mutations identified in each round were combined iteratively via site-directed mutagenesis and then tested for increased methanol tolerance. The best recombinant from each round was then used as the parent for the next round of error-prone PCR. After introduction of the disulfide bond (Dieselzyme 1) and 3 rounds of random mutagenesis with site-directed recombination (Dieselzymes 2–4), a mutant (Dieselzyme 4) with 13 amino acid changes (Table 1) was identified. Dieselzyme 4 retained more than 80% of its residual activity after incubation with 70% methanol for 16 hours and displayed further improved thermostability compared to the disulfide mutant Dieselzyme 1 (see below).

**Table 1:** List of mutations in engineered Dieselzymes

**Construct**	**Mutations present**
**Dieselzyme 1**	G181C/S238C*
**Dieselzyme 2**	G181C/S238C/**K208N**/**L64I**/**A70T**/**F225L**/**Q277L**
**Dieselzyme 3**	G181C/S238C/K208N/L64I/A70T/F225L/Q277L/**G202E**/**G266S**/**D270N**/**N17S**^#^
**Dieselzyme 4**	G181C/S238C/K208N/L64I/A70T/F225L/Q277L/G202E/G266S/D270N/N17S/**I255F**/**R33T**

Mutations in bold indicate the new mutations that were identified in each round of directed evolution. The additive effect of mutations was analyzed by combining mutations through site-directed mutagenesis. Only the final mutant from each round is shown. * - Construct used as parent for the initial round of directed evolution. # - N17S mutation arose spontaneously while combining the G202E, G266S, and D270N mutations.

### Characterization of thermostability and alcohol tolerance of evolved PML mutants

**Figure 4:**
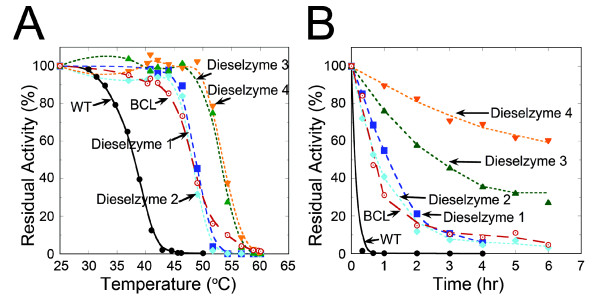
**Temperature induced inactivation of Dieselzymes.** (**A**) Inactivation as a function of temperature. Residual activity was measured after incubation for 1 hour at various temperatures. (**B**) Time course of thermal inactivation at 50°C. Results are the average of 3 independent experiments. Error-bars are omitted but are less than 5% in all cases. Lines are shown for clarity and represent the best-fit to the data points. WT PML (black circles), BCL (red open circles), Dieselzyme 1 (blue squares), Dieselzyme 2 (blue diamond), Dieselzyme 3 (green triangle), and Dieselzyme 4 (orange inverted triangle).

**Figure 5:**
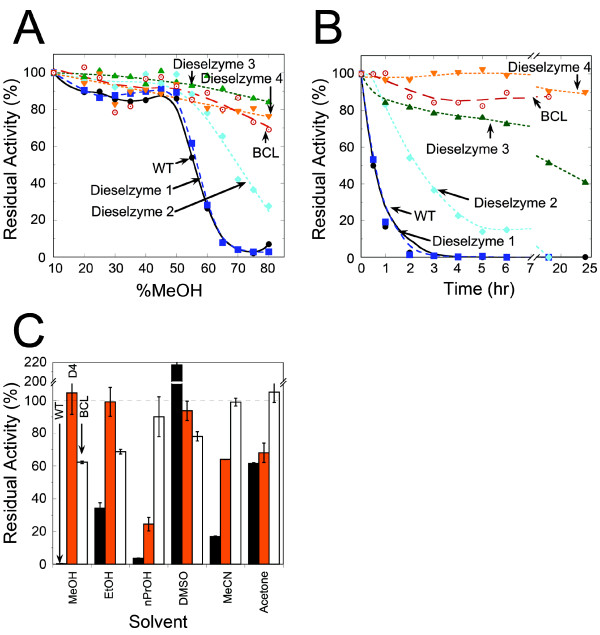
**Methanol induced inactivation of Dieselzymes.** (**A**) Methanol inactivation as a function of concentration. Samples were incubated for 2 hours and assayed after dilution to 10% methanol. Results are the average of 3 independent experiments. (**B**) Methanol inactivation as a function of time. Samples were incubated in 50% methanol for 24 hours. At indicated time points, samples were diluted to 10% methanol and assayed for residual activity. Error-bars are omitted but are less than 5% in all cases. Lines are shown for clarity only. Results are the average of 3 independent experiments. WT PML (black circles), BCL (red open circles), and Dieselzyme 1 (blue squares), Dieselzyme 2 (blue diamond), Dieselzyme 3 (green triangle), and Dieselzyme 4 (orange inverted triangle). (**C**) Inactivation profile of Wild-Type PML (black bar), Dieselzyme 4 (orange bar), and BCL (white bar) in response to various solvents. Enzymes were incubated for 24 hours in 70% solvent and diluted to 10% prior to assaying for residual activity. Results are the average of three independent experiments.

To evaluate the full extent of stabilization after random mutagenesis, the residual activity of wild-type PML was compared with each Dieselzyme variant as a function of temperature or methanol concentration as shown in Figures 4 and 5 respectively. The thermal inactivation profile for the disulfide bond mutant Dieselzyme 1 and Dieselzyme 2 are identical. However, in Dieselzyme 3 and Dieselzyme 4, the added mutations have a beneficial effect on thermostability with an apparent IT_1/2_ after 1 hr of 55°C for both Dieselzyme 3 and Dieselzyme 4, which is an increase of 17°C compared to wild-type and ~5°C higher than Dieselzyme 1 (Figures 1B, 4A). In addition, when the mutant lipases were incubated at a constant 50°C, the final mutant Dieselzyme 4 also showed a further improvement over Dieselzyme 3 with an inactivation half-life of ~7 hours (Figure 4B) which is more than a 30-fold improvement over wild-type (less than 15 min). Dieselzyme 3 and Dieselzyme 4 are also more resistant to thermal inactivation than the industrial lipase from *B*. *cepacia* described above.

In addition to thermostability, the methanol tolerance of PML was also significantly improved over four rounds of screening. The methanol tolerance of wild-type and mutant PMLs as a function of methanol concentration is shown in Figure 5A. Due to activation of PML in low concentrations of methanol (not shown), the residual activity was normalized to activity remaining after incubation with 10% methanol. Similar activation of related lipases with short-chain alcohols has been demonstrated recently [30]. When the wild-type or Dieselzyme 1 is incubated for two hours at increasing methanol concentrations, both enzymes retain 50% activity in 55% methanol and are completely inactivated at 70% methanol. In contrast, Dieselzyme 2 retained ~20% residual activity after incubation with 70% methanol while the Dieselzyme 3 and Dieselzyme 4 retain greater than 70% of their activity after incubation in 70% methanol.

While we were able to improve the absolute methanol tolerance of PML during a relatively short incubation, the actual goal was to develop a mutant lipase that had better long-term resistance to the presence of methanol. In order to determine if the mutant lipases were also more resistant to methanol inactivation over a long period of time, wild-type or mutant PML was incubated for 24 hours in the presence of 50% methanol. Figure 5B shows the residual activity of wild-type and mutant PML from the different rounds of screening after treatment with 50% methanol as a function of time. Clearly the long-term resistance was greatly improved by the screening regimen, with Dieselzyme 4 retaining roughly 90% activity after incubation with 50% methanol for 24 hours. The *B*. *cepacia* lipase that is currently used industrially for biodiesel synthesis shows a comparable level of tolerance under these conditions. As shown in Figure 5C, however, when we raise the concentration of methanol to 70%, Dieselzyme 4 retains essentially full activity after 24 hrs, but the *B*. *cepacia* lipase retains only ~60%.

### Other alcohols and water miscible solvents

To test whether the improvement in methanol tolerance also rendered Dieselzyme 4 more resistant to other water-miscible organic solvents, we incubated the enzymes in the presence of a variety of organic solvents at a concentration of 70% for 24 hours, and then assayed for residual hydrolysis activity (Figure 5C). In general, Dieselzyme 4 retained considerably more activity than the wild-type after incubation with organic solvents, with the exception of DMSO. DMSO surprisingly stimulates the wild-type PML enzyme, but has little effect on Dieselzyme 4.

We also compared the organic solvent tolerance of Dieselzyme 4 to the tolerance of the *B*. *cepacia* lipase. As shown in Figure 5C, Dieselzyme 4 is more tolerant than the *B*. *cepacia* lipase to the alcohols most commonly used in biodiesel synthesis, methanol and ethanol. The *B*. *cepacia* lipase is more tolerant to 1-propanol, acetonitrile and acetone, however, indicating that organic solvent tolerance is a complex property.

### Transesterifcation and recycling of immobilized PML

As our goal is to develop a stable lipase that is less prone to methanol induced inactivation during biodiesel synthesis, we tested the ability of Dieselzyme 4 to retain transesterification activity for many cycles over a long period of time. To monitor both transesterification activity and resistance to methanol induced inactivation during multiple rounds of synthesis, wild-type, Dieselzyme 4, and the industrial enzyme BCL were covalently immobilized onto hydrophobic oxirane functionalized beads. Covalent immobilization was used to ensure differences in activity were due to enzyme inactivation as opposed to loss by desorption. To monitor biodiesel synthesis, 200 μg lipase covalently immobilized on 250 mg beads were added to a mixture of 0.625 ml of 50% methanol and 1.5 ml of canola oil. This mixture provides a ~5:1 molar ratio of methanol to triacylglycerol oil or a ~1.6 molar ratio of methanol per ester bond. The final water content was ~1:4 (or 26% w/w) of the oil. To test the response of the enzymes to prolonged exposure to methanol, a small amount of immobilized enzyme beads were added such that there was less than 10% conversion in the first hour. A similarly low extent of conversion for all enzymes during the first few hours was used to ensure differences seen between enzymes were due to the time of exposure to methanol. For each cycle the progress of the reaction was monitored after 20 hours. For the first, fourth and seventh cycles, we also monitored the reaction progress at 2, 4 and 6 hours to observe initial rates. To restart the reaction at each cycle, the beads were separated by filtration and washed prior to reuse in a subsequent round of biodiesel synthesis. A water wash was performed to remove glycerol and residual methanol followed by a wash with hexanes to remove residual oil and fatty acid methyl esters.

**Figure 6:**
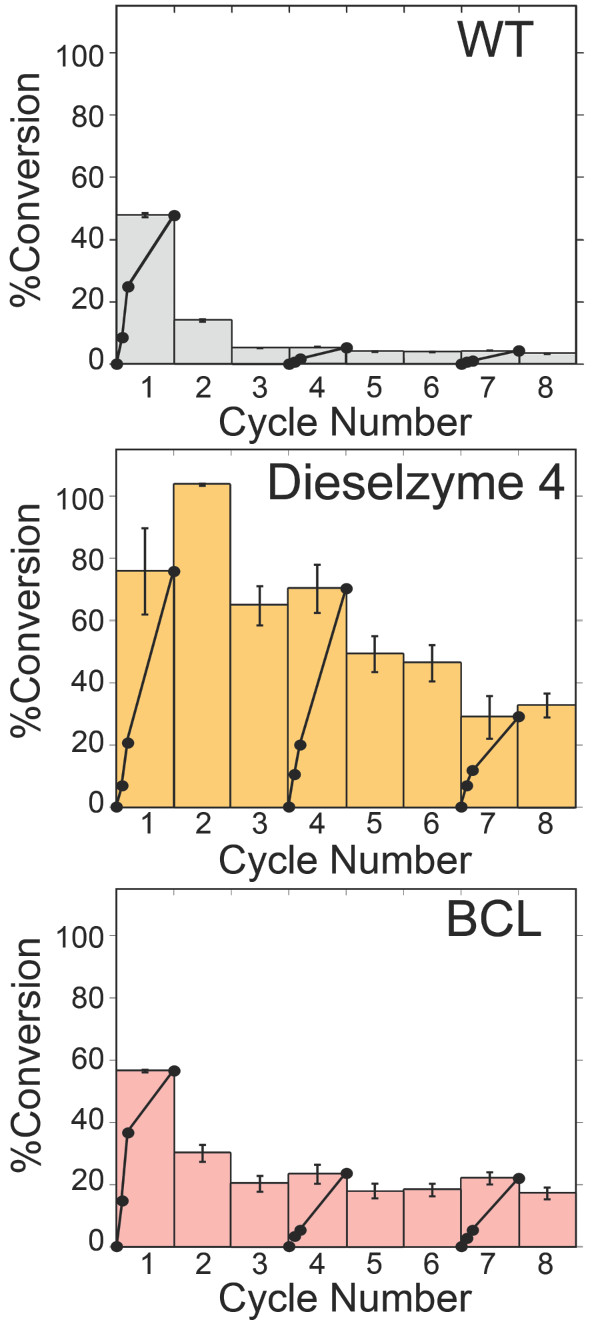
**Operational stability of covalently immobilized Wild-Type PML, Dieselzyme 4, and BCL.** For each, the same amount of initial activity was added such that >40% conversion was seen over the first four hours: 0.25 g immobilized WT PML; 0.25 g immobilized Dieselzyme 4, or 0.1 g BCL. For each round, the reaction conditions were: 1.5 mL canola oil, 312.5 μL methanol (5:1 MeOH:oil), 312.5 μL 0.1 M sodium phosphate pH 7.0 (20% v/v with oil); temperature 25°C; 180 rev/min. During cycles 1, 4, and 7, 100 μL samples were taken at 0, 2, and 4 hours to monitor rate of reaction. After each 20 hour cycle, beads were washed with buffer followed by hexanes prior to addition of fresh substrates and buffer. The data are the average of two experiments.

The results of multiple rounds of transesterification can be seen in Figure 6. During the first cycle the initial rate of biodiesel synthesis was linear and showed a similar rate of conversion for all three immobilized enzymes, with 24.7%, 20.6%, and 36.5% conversion to methyl esters for WT, Dieselzyme 4, and BCL respectively after 4 hours. After 4 hours the rate of synthesis decreased for both WT and BCL, such that only ~47.7% and ~56.4% total conversion was seen after 20 hours. In contrast, Dieselzyme 4 continued at a high rate of synthesis, converting ~76% of the canola oil to biodiesel within 20 hours. It is unclear why conversion by Dieselzyme 4 did not reach 100% in the first round. However, it is obvious that even though there was higher initial BCL activity, Dieselzyme 4 was able to convert more oil over the longer incubation.

The improved methanol tolerance of Dieselzyme 4 becomes even more apparent when the immobilized enzymes are reused in a second cycle. After a single reuse, the wild-type PML lost nearly all of its ability to catalyze transesterification with less than 15% of the canola oil being converted to biodiesel in the second round. Similarly, BCL also showed a decreased yield of biodiesel with only 30% conversion of TAG to FAME in the second round. In contrast, Dieselzyme 4 retained complete activity in the second round. Overall, the rate of inactivation of Dieselzyme 4 was significantly slower than that of BCL as Dieselzyme 4 retained nearly the same amount of activity after round 4 compared to round 1 and still converted 50% of canola oil to biodiesel after the fifth cycle (compared to only 18% for BCL after cycle 5). These results show that not only is Dieselzyme 4 active for transesterification, but Dieselzyme 4 is significantly more resistant to methanol induced inactivation during biodiesel synthesis compared to wild-type PML and even outperforms BCL.

### Productivity of Dieselzyme 4

Fjerbaek et al. define productivity in terms of kg of biodiesel produced per kg of catalyst [9]. Their cost analysis suggests that a 23-fold improvement in lipase productivity from the current high water mark of 7400 kg/kg to ~170,000 kg/kg would make enzyme catalyzed biodiesel production cost neutral with the base catalyzed reaction (not including the many advantages of lipase catalysis). To measure the potential productivity of Dieselzyme 4, we used milder conditions than the recycling test above, while still employing single additions of methanol. 200 μg of purified enzyme was immobilized on 0.5 g of oxirane functionalized beads and used to convert 5 mL (4.5 g) of refined oil to biodiesel using 2 mL of 40% methanol (equivalent to 4:1 molar ratio of methanol:oil and 25% water (v/v water/oil)). As shown in Additional file 3: Figure S3, greater than 50% of the input oil was converted to biodiesel in the first cycle. Similar to the recycling experiments above, Dieselzyme 4 retains ~50% of the initial activity after 6 cycles (1.1 g biodiesel produced in cycle 5 compared to 2.4 g biodiesel produced in cycle 1). Overall, the productivity of immobilized Dieselzyme 4 is ~46,000 kg/kg after 6 cycles. Assuming an exponential decay of activity, the productivity of Dieselzyme 4 is projected to plateau at ~82000 kg/kg (Additional file 3: Figure S3). Thus, the productivity of Dieselzyme 4 is between 46,000 and 82,000 kg/kg, which is within a 2- to 4-fold improvement in activity, longevity, process enhancements or enzyme production costs of being cost equivalent with base catalysis.

### Structural basis for evolved methanol tolerance

**Figure 7:**
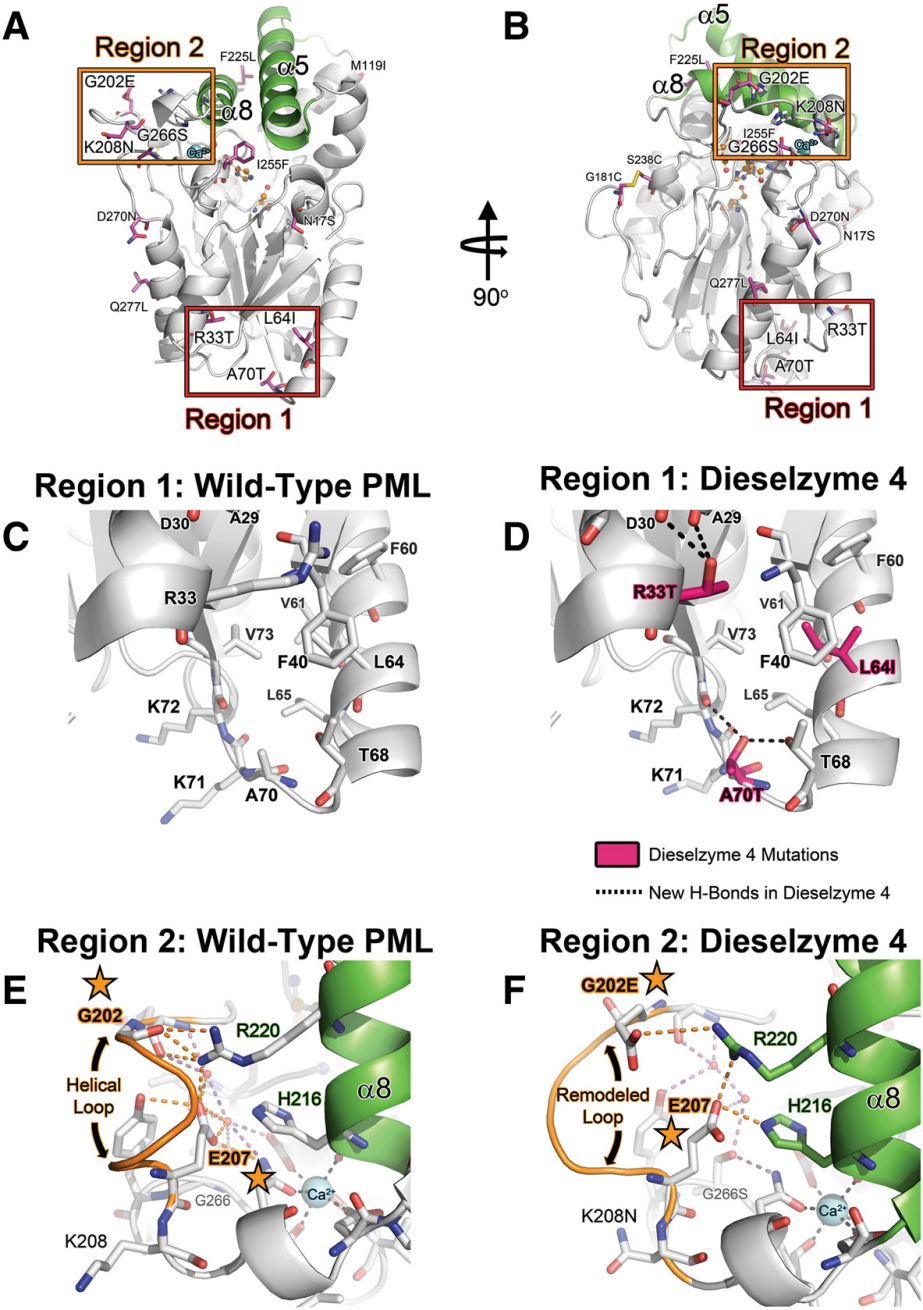
**Structure of PML mutant Dieselzyme 4.** (**A**) Cartoon representation of Dieselzyme 4. The locations of clustered mutations described in the text, designated Region 1 and Region 2, are highlighted with a red and orange box respectively. Mutations in Dieselzyme 4 are labeled and shown as sticks. Lid helices α5 and α8 are shown for clarity and colored green. The catalytic triad is shown as orange ball and stick. (**B**) 90° rotation of (**A**). (**C**) Zoom in of Region 1 as seen in the wild-type PML structure. (**D**) Zoom in of Region 1 as seen in the Dieselzyme 4 structure. Mutations are highlighted as magenta sticks. New H-bonds present in Dieselzyme 4 are shown as dashed black lines. (**E**) Zoom in of Region 2 as seen in the wild-type PML structure. The “helical loop” between residues 202 and 207 is highlighted orange. Potential H-bonds between residues R220 and H216 from helix α8, the loop region, and crystallographic waters are shown as orange dashes. The bound calcium is shown as a cyan sphere. (**F**) Zoom in of Region 2 as seen in the Dieselzyme 4 structure. The remodeled “helical loop” between residues 202 and 207 is highlighted in orange. Residues G202E and E207 that form potential new salt-bridges with R220 and/or H216 from helix α8 are highlighted with a star. Other H-bonds are shown as red dashes. The bound calcium is shown as a cyan sphere.

The structure of Dieselzyme 4 was determined by x-ray crystallography to examine the effects of the mutations on the PML structure. Besides the introduced disulfide bond discussed above, 11 new mutations arose from in vitro evolution. An analysis of the crystal structure of Dieselzyme 4 suggests that 6 mutations cluster into two distinct areas, (Region 1 and Region 2) that may be important for the improvement in methanol tolerance (Figure 7A and 7B). The other 5 mutations may be neutral with respect to methanol tolerance because they are located on the surface (F225L, D270N, Q277L) or in regions that do not lead to obvious differences (N17S, I255F).

Region 1 comprises the mutations R33T, L64I, and A70T that cluster on or near helices α1 and α2 at opposite end of the protein from the lid region (Figure 7C and 7D). Well-defined electron density was seen for all three of the new side chains in this region (Additional file 2: Figure S2B). Mutation of leucine to isoleucine at position 64 provides more interactions with side-chains F40 and F60, possibly improving packing to stabilize helix α2. Distance measurements of the A70T mutation, which arose in the first round of error-prone PCR (Dieselzyme 2), shows that the hydroxyl from the threonine forms a new H-bond interaction with T68 and the backbone carbonyl of K72 (Figure 7D). Similarly, the R33T mutation that arose after the final round of error-prone PCR (see Dieselzyme 4) forms new H-bond interactions between the threonine hydroxyl and the backbone carbonyls from A29 and D30. In polar organic solvents such as methanol, it has been hypothesized that inactivation and unfolding of proteins is due to a combination of stripping of water from the protein surface and hydrophobic collapse of buried non-polar regions due to the destabilizing effect of aliphatic alcohols on tertiary interactions [31,32]. It is possible that the L64I mutation provides improved packing interactions that counteract the hydrophobic collapse of Region 1 in response to methanol while the R33T and A70T provide stabilizing H-bonds. Due to the lower dielectric constant of methanol, these introduced H-bonds may be further strengthened [33]. It has also been reported for CALB lipase [34], phospholipase A1 [35], and metalloprotease [36], that the introduction of polar residues can lead to increases in solvent tolerance.

Region 2 mutations (Figure 7E and 7F) cluster near the Ca^2+^ binding site, and cause an unexpected remodeling of the loop between residues 200 and 208. The introduction of polar side chains may also stabilize the Ca^2+^ binding site and provide additional polar interactions between the loop and lid helix α8 that may resist the destabilization due to methanol. While the density of the remodeled loop is not as well-defined as the rest of the protein (Additional file 2: Figure S2C), there is sufficient electron density to provide a possible rationale for the observed improvement in methanol tolerance. Loop mutations G202E, K208N, and G266S and are located close to the Ca^2+^ binding site which is essential for lipase stability and activity. The most striking change in the loop is a direct result of the G202E mutation. The introduced glutamate side chain at position 202 is positioned ~4 Å from R220 in an orientation that could form a new salt-bridge which may provide stability in the low dielectric methanol environment. In order to make this potential new salt-bridge, the helical region between residues 203 and 207 seen in wild-type PML partially unravels and may increase flexibility of the loop region to allow new interactions to occur. For instance, the increased flexibility due to unraveling of the helix between 203 and 207 may allow E207 to move and form a new salt-bridge with R220 and conserved H216. However, the electron density surrounding R220 is split, suggesting that R220 is in at least two conformations: one that can interact with E207 and one that potentially interacts with the introduced G202E mutation (Figure 7F). Additionally, while the K208N mutation is well defined and does not alter the backbone or side chain conformation at position 208, it is possible that removal of the charged Lys also increases the flexibility of the loop region, facilitating the new interactions made by the G202E mutation and E207 that stabilize this region in the presence of methanol.

Interestingly, in addition to the polar residue insertion (K208N/G202E) and loop remodeling described above, the G266S mutation forms another new polar interaction, albeit with a crystallographic water and the side chain N210 that directly coordinates the Ca^2+^. It has been proposed that a major effect of alcohols on proteins is the stripping of water from the protein surface leading to destabilization [37]. Previous experiments with the homologous lipases from *B*. *glumae* have also indicated that the bound Ca^2+^ plays an important role in lipase stability [38]The introduction of a new polar interaction from G266S may help keep water bound and further stabilize the bound Ca^2+^ in Dieselzyme 4 by providing a new H-bond to N210. Together, the polar mutations in Region 2 appear to provide stabilizing interactions with the lid helix α8 (through R220 and H216) and to potentially stabilize the Ca^2+^ binding site that may be sensitive to the destabilizing effects of methanol and other alcohols.

## Conclusion

We have employed directed evolution for the first time to improve properties of a lipase for biodiesel production. Unlike the *B*. *cepacia* lipase, PML is highly expressed in an active form in *E*. *coli*, making it a viable platform for engineering efforts. The evolved PML enzyme, Dieselzyme 4, catalyzes efficient synthesis of biodiesel even in the presence of a high concentration of water and has better methanol tolerance and heat stability than the top industrially used lipase from *B*. *cepacia*. The success of the screening protocol and identification of regions on the enzyme central to improved stability makes Dieselzyme 4 a viable platform for further engineering by further directed evolution, perhaps by targeted methods such as CAST [39] or ISM [40] to develop better catalysts for biodiesel production in the future.

## Methods

### Materials

All chemicals were of analytical grade or better. 4-nitrophenyl palmitate, and Triton X100 were from Sigma. 1-naphthyl palmitate and Fast Blue B were from MPI Biochemicals. All other solvents were from Fluka. Amano lipase PS (*Burkholderia cepacia*) was purchased from Sigma and was purified before use using a HiTrapQ column (GE life sciences) and dialyzed into 20 mM Tris-Cl pH 7.5, 0.1 M NaCl. Primers for cloning and mutagenesis were ordered from Valuegene. Refined canola oil used for transesterification was from the local market.

### Construction of mutants by site-directed mutagenesis

Site-directed mutagenesis was performed using the Quick-change Site Directed Mutagenesis Kit (Stratagene) according to the manufacturer’s directions. For generation of double mutants, a modified megaprimer method was used [41]. Briefly, in an initial PCR, a forward and reverse mutagenic primer, each containing a different mutation, was used to amplify a short segment of the PML gene using Taq Hot-Start Supermix (Denville) to generate a megaprimer. In a second PCR reaction, 5 μL of the initial PCR was mixed with 30 ng plasmid template, 0.2 μM dNTP, Pfu HotStart II Reaction Buffer, and 2.5 U of Pfu HotStart Fusion II DNA Polymerase (Stratagene) in a 50 μL reaction. The reaction was cycled as for the QuikChange reaction. The resulting PCR product was digested with *DpnI* and used to transform BL21Gold(DE3) or XL10Gold directly.

### Construction of PML mutant library by error-prone PCR

The wild-type lipase gene from *Proteus mirabilis* was cloned into a pET28a vector as previously described and used as template for error-prone PCR. Random mutagenesis was performed using the Genemorph II Kit (Stratagene) according to the manufacturer’s instructions to ensure a 1-2% error rate per gene (1–5 amino acid changes). Primers which flank the 5’*NdeI* and 3’*EagI* restriction sites were used for amplification. An appropriate amount of DNA template was used to generate between 1 and 5 mutations per 1 kb. Briefly, 0.1 ng template was mixed with 0.2 mM each dNTP, 0.2 μM each primer, 1X Mutazyme II Buffer, and 2.5 U Mutazyme II in 50 μL. The PCR was incubated at 95°C for 2 minutes followed by 45 cycles of 95°C for 30 s, 58°C for 30 s, 72°C for 1 min, with a final extension at 72°C for 10 minutes. The resulting PCR product was purified using a QIAquick spin column (Qiagen) and digested overnight with *NdeI* and *EagI* (NEB) at 37°C. The digested product was gel purified and ligated into *NdeI*/*EagI* digested pET28 using T4 DNA ligase (NEB) for 16 hours at 16°C. The resulting library was directly transformed into chemically competent BL21Gold(DE3) and plated on LB-agar containing 50 μg/mL kanamycin for screening and analysis. To confirm the desired 1-2% error rate per gene, plasmid was isolated from 20 colonies and sequenced (Genewiz).

### Library screening for MeOH tolerance

Screening for improved methanol tolerance was accomplished by a colony lift screening protocol with a PML mutant library expressed in BL21Gold(DE3). Following overnight incubation at 37°C, transformants (~600/plate) were lifted onto sterile filter circles (Whatman 410) and placed colony-up on a LB-agar plate containing 50 μg/mL kanamycin and 1 mM IPTG for 2–3 hours at 18°C to induce protein expression. The filter was then immersed in lysis solution (50 mM sodium phosphate pH 7.5, 0.1 M NaCl, 0.1% Triton X100, 1 mg/mL lysozyme) at 25°C for 1 hour. Lysis solution was decanted and replaced by MeOH solution containing 0.1% Triton X100 and incubated at a selected temperature for a desired amount of time. After incubation, the MeOH solution was decanted and the filters were developed by overlaying with 1 mM 1-naphthyl palmitate, 3 mM Fast Blue B, 0.5% Triton X100 dispersed in 0.5% agar. After 10 minutes, mutants displaying residual activity were identified by formation of a purple color due to the azo dye formed between 1-naphthol and Fast Blue B (Figure 3). The corresponding colony was then isolated from the master plate for validation and further characterization.

For validation, positives from the filter screen were grown in 10 mL LB containing 50 μg/mL kanamycin to OD_600_ of 0.6 and protein expression was induced with 0.5 mM IPTG for 16 hours at 18°C. The cells were pelleted (6K rcf × 30 min; Sorvall GS-3 rotor), resuspended in buffer (50 mM sodium phosphate pH 7.5, 0.1 M NaCl, 5 mM imidazole), lysed by sonication (5 × 30 s pulse) and clarified by centrifugation (20K rcf × 30 min; Sorvall SS-34 rotor). To validate, 50 μL of the supernatant was incubated with 70% MeOH for 1 hour and then diluted to 10% MeOH prior to being assayed with 1 mM *p*-nitrophenyl palmitate (pNPP). Residual activity was defined as the activity compared to a sample incubated at 10% MeOH for 1 hour. Mutations were confirmed by sequencing (Genewiz) and the beneficial mutations were combined by site-directed mutagenesis and reassayed for improved MeOH tolerance. The best performing combined mutants were then used as parents for subsequent rounds of directed evolution.

### Expression and purification of WT and mutant PMLs

Over-expression of wild-type and mutant PML was carried out in *E. coli* BL21Gold(DE3) (Agilent). Single transformants were transferred to 2 L of Luria-Burtani (LB) media containing kanamycin for incubation at 37°C and grown to an OD_600_ of 0.6. Protein expression was induced with 0.5 mM IPTG at 16°C for 16 hours. Cells were harvested by centrifugation and purified by Ni-NTA chromatography (Qiagen) as described previously. Wild-type and mutant PMLs were then dialyzed into 20 mM Tris–HCl pH 7.5 containing 100 mM NaCl and flash frozen as droplets in liquid N_2_ prior to storage at −80°C.

### Enzyme kinetics and characterization

Kinetic assays were carried out in 96 well microtiter plates (Grenier Bio-One) with purified protein. For kinetic analysis a stock solution of 86.13 mM pNPP was prepared in 1:1 Acetonitrile:Triton X100. The initial rate of conversion of pNPP to p-nitrophenol was monitored at 405 nm over the first minute using a plate reader (SpectraMax M5, Molecular Devices). For temperature or methanol incubation studies, the residual activity was assayed using 1 mM pNPP in 50 mM phosphate pH 7.0. For temperature inactivation, 60 μL of 150 nM enzyme was incubated in 50 mM phosphate pH 7.0 in thin walled PCR using a PCR cycler (Mastercycler ProS, Eppendorf). The residual activity remaining as a function of incubation for 1 hour at various temperatures or as a function of time at 50°C was determined by hydrolysis of pNPP. Activity was normalized to activity at 25°C. For methanol tolerance, 1.5 μM enzyme was incubated with 50 mM phosphate pH 7.0 containing various concentrations of methanol. After 2 hours, the enzyme was diluted 10-fold so that the final methanol concentration was 10% and the final enzyme concentration was 150 nM. For inactivation by methanol over time, the enzyme was incubated in 50% methanol at 25°C. At various time points, 10 μL aliquots were diluted 1:10 with 90 μL 5.5% methanol (10% final) and assayed for residual activity compared to incubation with 10% methanol as described above.

### Immobilization and transesterification

Purified *B. cepacia*, PML, and mutant lipases (Dieselzymes) were covalently immobilized on hydrophobic Immobead 350 oxirane functionalized beads (ChiralVision) prior to use. Beads (0.25 g) were washed once with 10 mL methanol followed by two washes with 10 mL 0.1 M sodium phosphate pH 7.0. The buffer was decanted and 1 mL enzyme at 0.2 mg/mL in 20 mM Tris–HCl pH 7.5, 0.1 M NaCl was added. Immobilization was allowed to proceed for 16–20 hours at 25°C. All 3 enzymes studied were immobilized to a similar degree (≥95%) as monitored by OD_280nm_ and by residual activity remaining in the supernatant.

After immobilization, the buffer was decanted and the beads were used for transesterification without further modification. For the synthesis reaction, 0.625 mL of 50% methanol (1:1 methanol:0.1 M phosphate pH 7.0) was added to the beads followed by 1.5 mL refined canola oil. The solution was gently mixed with a vortex and then placed on a shaker at 200 rpm at 25°C. An initial reaction was performed with 0.1 g beads to establish the transesterification rate for each construct. For the recycling experiment, an appropriate amount of beads (0.25 g, 0.25 g, and 0.08 g of wild-type PML, Dieselzyme 4, and BCL respectively) were added such that ~5-10% conversion was reached in the first hour. After each 20 hour cycle, 10 μL aliquots of the oil layer were taken for analysis by gas chromatography (below). To monitor the effect of reuse on initial rate, 10 μL were taken at 2 and 4 hours during cycles 1, 4, and 7. For reuse, the beads were recovered by filtration, washed with 5 mL buffer followed by 5 mL hexanes and then allowed to dry before adding fresh methanol, buffer, and oil.

### Quantification of fatty acid methyl esters

The extent of transesterification was monitored by gas chromatography (GC). For analysis, 10 μL samples were diluted with 1 mL hexane spiked with 0.5 mg/mL methyl heptadecanoate (internal standard). 1 μL of the samples at a split ratio of 50:1 were analyzed on an Agilent 5890 Series II GC with flame ionized detector using an HP-INNOWax column (0.25 mm × 30 m, Agilent). The carrier gas was helium with a flow rate of 5 mL/min. The oven temperature was kept at 200°C for 3 min and then raised to 230 at 5°C/min then to 250 at 20°C/min and held at 250°C for 9 min. The injector and detector temperatures were kept at 230 and 330°C respectively. The percent conversion was determined by comparison to a biodiesel sample prepared from refined canola oil using a large excess of free *Burkholderia cepacia* lipase at a 5:1 MeOH:oil ratio in the presence of 5% water as described previously [23].

### Crystallization, structure determination, and refinement of mutant PML

Crystallization trials for Dieselzyme 4 were performed using purified His-tagged lipase with no additives. Drops were generated by mixing 2 μL purified protein at 9 mg/mL with 2 μL well solution. Large crystals of the mutant PML formed in many conditions between one day and two weeks. One condition, Qiagen PACT condition #38 (1X MMT pH 5, 20% PEG 1500), was optimized and gave large crystals (0.2×0.2×0.2 mm) within one week. Prior to data collection, crystals were soaked in crystallization well solution plus 15% glycerol and flash frozen in liquid nitrogen. Data were collected in house on a Rigaku FRE + x-ray generator equipped with an ADSC Quantum 4 CCD detector at 100 K. Diffraction images were indexed, integrated, and scaled with Denzo and Scalepack. Initial phases were determined by Molecular Replacement using PHASER in CCP4i. The wild-type PML (PDBID 4GW3) was used as the search model. The resulting model was further refined via iterative rounds of model building and refinement in COOT and Refmac5 over the resolution range 50–1.8 Å. The final model was deposited in the Protein Data Bank with PDBID 4HS9. Data statistics can be found in Additional file 4: Table S1.

## Abbreviations

FAME: Fatty acid methyl ester; TAG: Triacylglycerol; MeOH: Methanol; EtOH: Ethanol; FFA: Free fatty acids; DMSO: Dimethylsulfoxide; DMF: Dimethylformamide; MeCN: Acetonitrile; PML: *Proteus mirabilis* lipase; BCL: *Burkholderia cepacia* lipase; CALB: *Candida Antarctica* lipase B; PCR: Polymerase chain reaction; LB: Luria-burtani media; IPTG: Isopropyl-β-D-1-thiogalactopyranoside; pNPP: *Para*-nitrophenyl palmitate; CAST: Combinatorial active-site saturation test; ISM: Iterative saturation mutagenesis.

## Competing interests

The authors declare that they have no competing interests.

## Authors’ contributions

TPK designed experiments, carried out the biochemical experiments, solved the crystal structures, and drafted the manuscript. BS, DMC, and GLH carried out screening and mutagenesis experiments. MB participated in the analysis of results. JUB conceived of the study, and participated in its design and coordination and helped to draft the manuscript. All authors read and approved the final manuscript.

## Supplementary Material

Additional file 1: Figure S1 Thermal inactivation of Dieselzyme 1 by incubation at 50°C as a function of time. Results shown are an average of 3 independent experiments. Error-bars are omitted but are less than 5% in all cases. Lines are shown for clarity and represent the best-fit to the data points. WT PML (black circles), BCL (red open circles), and Dieselzyme 1 (blue squares). Click here for file

Additional file 2: Figure S2 Electron density of the Dieselzyme 4 crystal structure in the vicinity of (A) The introduced disulfide bond as a result of the G181C/S238C mutation (B) Region 1 and (C) Region 2 mutations. The 2Fo-Fc map is shown contoured to 1 σ. Click here for file

Additional file 3: Figure S3 Productivity of Dieselzyme 4. The enzyme was covalently immobilized on oxirane beads. The amount (g) of product produced in each round is plotted versus cycle number (black circle). The black line represents an exponential fit assuming a first order decay. The cumulative amount of biodiesel produced at the end of each round is also shown (red diamonds). The projected cumulative total biodiesel produced, estimated from the exponential decay, is shown as a red dashed line. Each reaction consisted of 200 μg purified enzyme immobilized on 0.5 g beads, 4.5 g canola oil, 2 mL 40% aqueous methanol. The reactions were incubated for 24 hours at 25°C on a rotary shaker at 200 rpm. The reactions were performed in duplicate and the results quantified by gas chromatography. Click here for file

Additional file 4: Table S1. Crystallization, data collection and refinement statistics. Click here for file
