# Supplementary figures and images for: Dieselzymes: development of a stable and methanol tolerant lipase for biodiesel production by directed evolution
Source: Biotechnol Biofuels. 2013 May 7;6:70. doi: 10.1186/1754-6834-6-70 (PMC3670234; doi:10.1186/1754-6834-6-70)

Residual Activity (%)

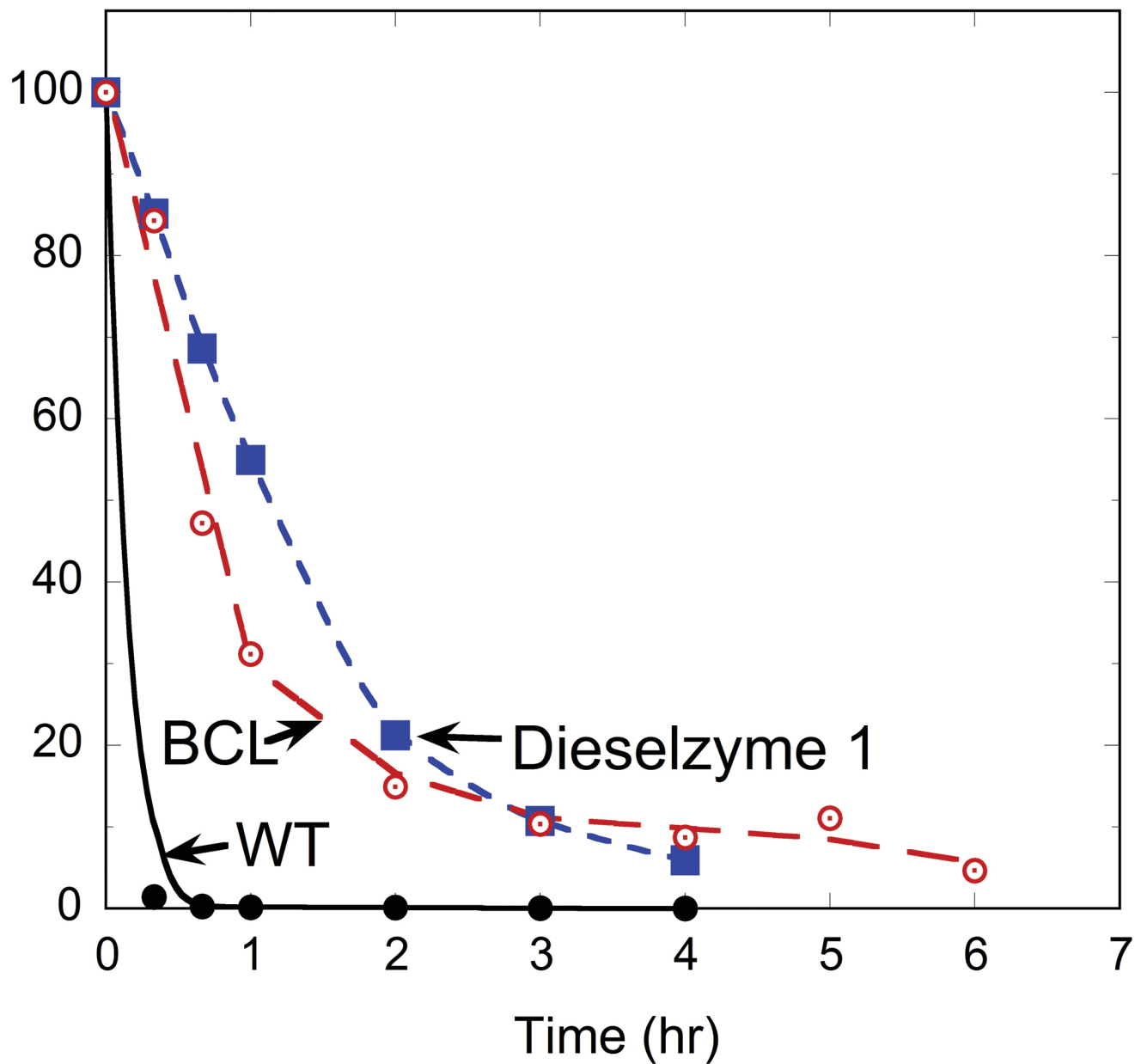

Supplement: Additional file 1: Figure S1 — Thermal inactivation of Dieselzyme 1 by incubation at 50°C as a function of time. Results shown are an average of 3 independent experiments. Error-bars are omitted but are less than 5% in all cases. Lines are shown for clarity and represent the best-fit to the data points. WT PML (black circles), BCL (red open circles), and Dieselzyme 1 (blue squares). [file 1754-6834-6-70-S1.pdf]

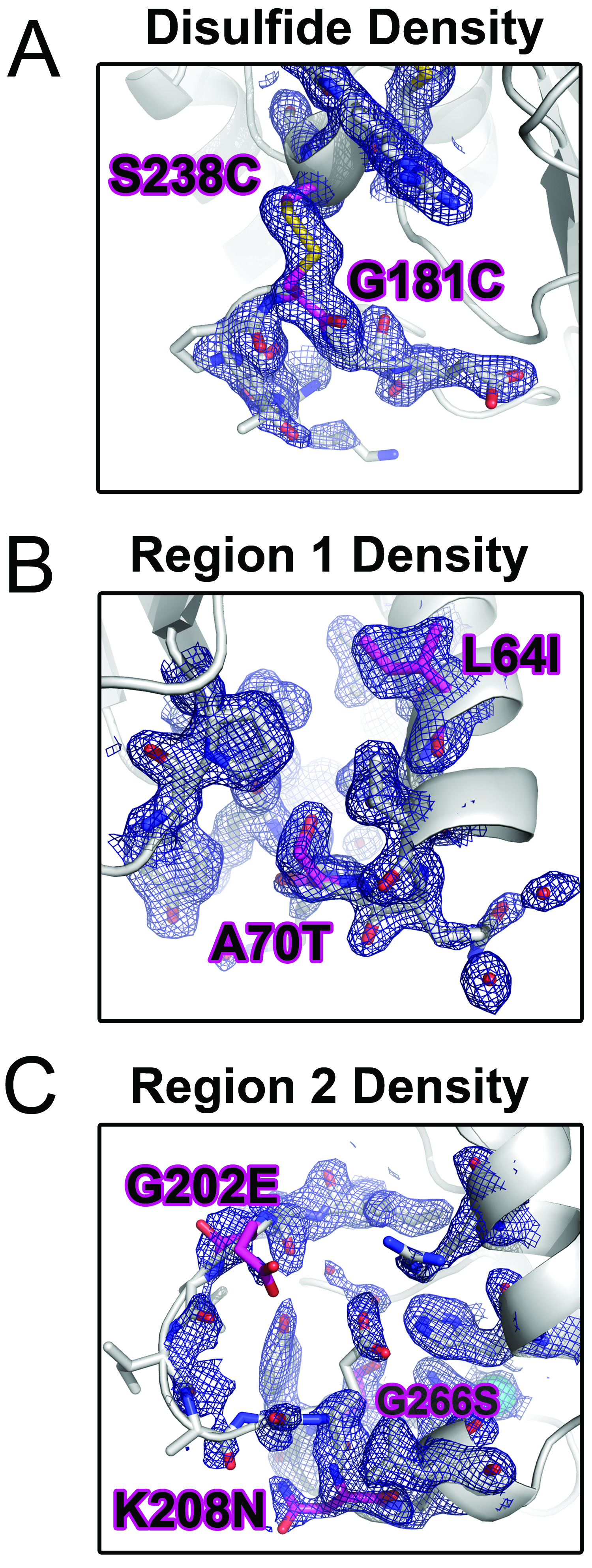

Supplement: Additional file 2: Figure S2 — Electron density of the Dieselzyme 4 crystal structure in the vicinity of (A) The introduced disulfide bond as a result of the G181C/S238C mutation (B) Region 1 and (C) Region 2 mutations. The 2Fo-Fc map is shown contoured to 1 σ. [file 1754-6834-6-70-S2.tiff]

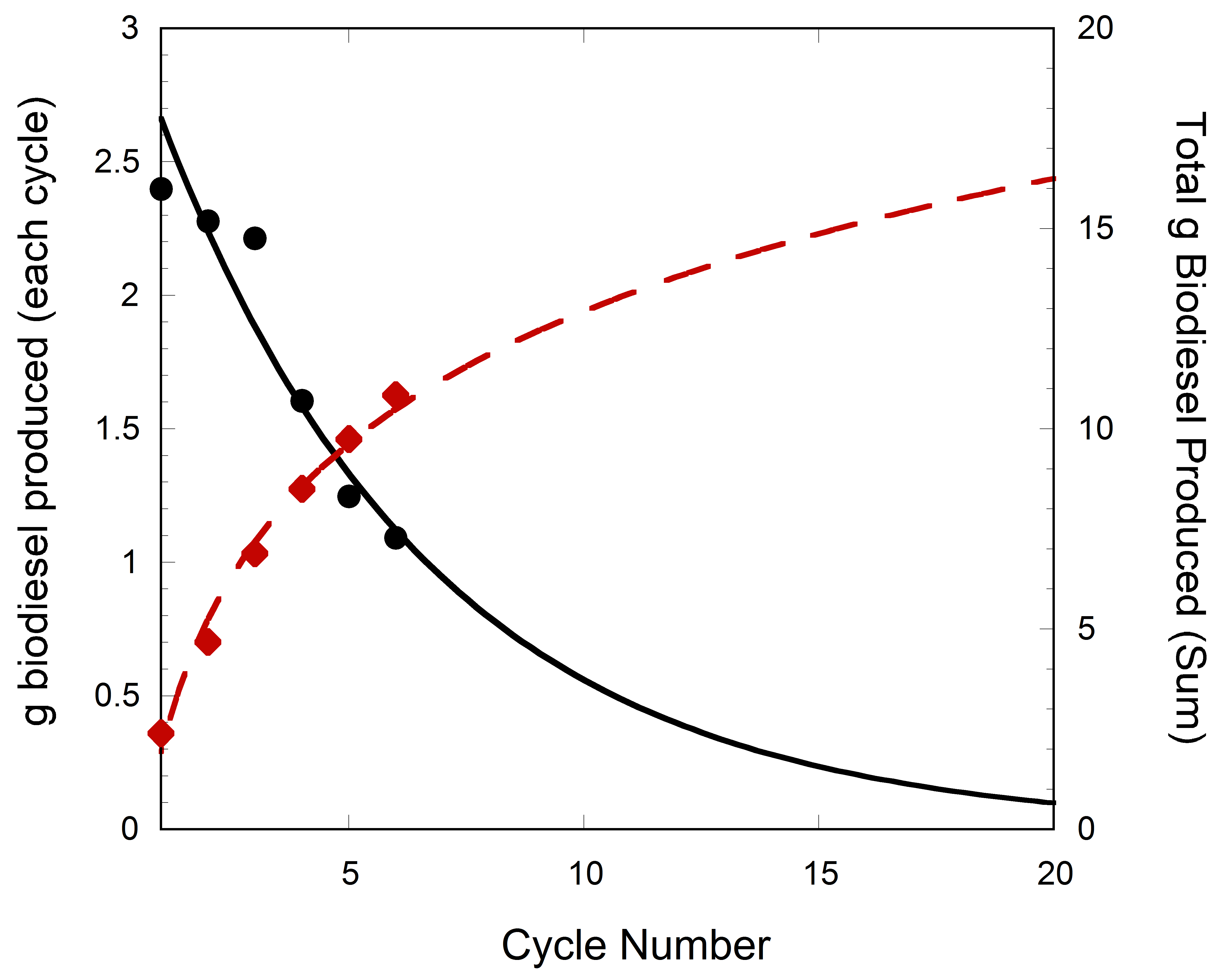

Supplement: Additional file 3: Figure S3 — Productivity of Dieselzyme 4. The enzyme was covalently immobilized on oxirane beads. The amount (g) of product produced in each round is plotted versus cycle number (black circle). The black line represents an exponential fit assuming a first order decay. The cumulative amount of biodiesel produced at the end of each round is also shown (red diamonds). The projected cumulative total biodiesel produced, estimated from the exponential decay, is shown as a red dashed line. Each reaction consisted of 200 μg purified enzyme immobilized on 0.5 g beads, 4.5 g canola oil, 2 mL 40% aqueous methanol. The reactions were incubated for 24 hours at 25°C on a rotary shaker at 200 rpm. The reactions were performed in duplicate and the results quantified by gas chromatography. [file 1754-6834-6-70-S3.tiff]
