# Supplementary material for: Dieselzymes: development of a stable and methanol tolerant lipase for biodiesel production by directed evolution
Source: Biotechnol Biofuels. 2013 May 7;6:70. doi: 10.1186/1754-6834-6-70 (PMC3670234; doi:10.1186/1754-6834-6-70)
Supplement: Additional file 4: Table S1. — Crystallization, data collection and refinement statistics. [file 1754-6834-6-70-S4.docx]

Supplemental Information

**Table S1.** Crystallization, data collection and refinement statistics

|  | Dieselzyme 4 |
| --- | --- |
| **A. Crystallization** | 1X MMT buffer (1:2:2 – DL-malic acid:MES:Tris base) pH 5.0 20% PEG1500 |
| **B. Crystallographic Data** |  |
| Space Group | P2_1_2_1_2_1_ |
| Cell Dimension (Å) | 48.1, 54.8, 96.0 α,β,γ = 90º |
| Resolution (Å) | 1.8 |
| Completeness % (last shell) | 96.6 (97) |
| I/σ(I) (last shell) | 20.70 (5.43) |
| Redundancy | 5.9 (5.6) |
| R_merge_ % (linear/square) | 6.8/8.1 (29.9/28.0) |
| **C. Refinement** |  |
| Resolution (Å) | 47.63-1.80 (1.84-1.80) |
| No. Reflections | 22136 (1672) |
| No. Protein Atoms | 2233 |
| No. Ligand/Ion Atoms | 47 |
| No. Waters | 116 |
| R_work_/Rfree % | 17.1/20.5 (26.1/31.5) |
| B-factor Protein | 7.14 |
| B-factor Ligand/Ion | 67.2 |
| B-factor Water | 37.8 |
| **D. Geometry** |  |
| RMS Bonds (Å) | 0.019 |
| RMS Angles (deg°) | 1.976 |
| Ramachandran Plot (%) |  |
| Most Favored | 91.3 |
| Favored | 7.5 |
| Generously Allowed | 1.2 |
| Disallowed | 0.0 |

**Supplemental Figure 1**: Thermal inactivation by incubation at 50 ºC as a function of time. Results shown are an average of 3 independent experiments. Error-bars are omitted but are less than 5% in all cases. Lines are shown for clarity and represent the best-fit to the data points. WT PML (black circles), BCL (red open circles), and Dieselzyme 1 (blue squares)

**Supplemental Figure 2:** Electron density of the Dieselzyme 4 crystal structure in the vicinity of (A) The introduced disulfide bond as a result of the G181C/S238C mutation (B) Region 1 and (C) Region 2 mutations. The 2Fo-Fc map is shown contoured to 1 σ.

**Supplemental Figure 3:** Productivity of Dieselzyme 4. The enzyme was covalently immobilized on oxirane beads. The amount (g) of product produced in each round is plotted versus cycle number (black circle). The black line represents an exponential fit assuming a first order decay. The cumulative amount of biodiesel produced at the end of each round is also shown (red diamonds). The projected cumulative total biodiesel produced, estimated from the exponential decay, is shown as a red dashed line. Each reaction consisted of 200 μg purified enzyme immobilized on 0.5 g beads, 4.5 g canola oil, 2 mL 40% aqueous methanol. The reactions were incubated for 24 hours at 25 ºC on a rotary shaker at 200 rpm. The reactions were performed in duplicate and the results quantified by gas chromatography.
